# Comprehensive Analysis of the Proteome of *S. cerevisiae* Wild-Type and *pdr*5Δ Cells in Response to Bisphenol A (BPA) Exposure

**DOI:** 10.3390/microorganisms13010114

**Published:** 2025-01-08

**Authors:** Valentina Rossio, Joao A. Paulo

**Affiliations:** Department of Cell Biology, Harvard Medical School, Boston, MA 02115, USA

**Keywords:** *S. cerevisiae*, bisphenol A (BPA), TMT proteomics, proteome, stress, Astral

## Abstract

Bisphenol A, an endocrine-disrupting compound, is widely used in the industrial production of plastic products. Despite increasing concerns about its harmful effects on human health, animals, and the environment, the use of BPA has been banned only in infant products, and its effects on cellular processes are not fully understood. To investigate the impact of BPA on eukaryotic cells, we analyzed the proteome changes of wild-type and *PDR5*-deleted *S. cerevisiae* strains exposed to different doses of BPA using sample multiplexing-based proteomics. We found that the ABC multidrug transporter Pdr5 plays an important role in protecting yeast cells from BPA toxicity, with its absence significantly sensitizing cells to BPA. BPA inhibited yeast growth in a dose-dependent manner, with a more pronounced effect in *PDR5*-deleted cells. Proteomic analysis revealed that BPA induces widespread dose-dependent changes in protein abundance, including the upregulation of metabolic pathways such as arginine biosynthesis and the downregulation of mitochondrial proteins. Additionally, we observed markers of cellular stress induced by BPA by identifying multiple stress-induced proteins that were upregulated by this compound. As cellular processes affected by BPA have been shown to be evolutionarily conserved, these insights can advance our understanding of BPA’s cellular impact and its broader effects on human health.

## 1. Introduction

First synthesized in 1891, bisphenol A (BPA), or 4,40-dihydroxy-2,2-diphenylpropane, has become a central component of modern industrial chemistry. BPA is widely used, particularly in the production of plastics, and it is present in everyday items such as food packaging, baby bottles, toys, and electronics [1,2]. However, its safety has been a subject of controversy as an unexpected discovery altered its perception in the early 1990s, providing evidence that BPA could leach from consumer products. Researchers discovered an estrogenic compound leaching from autoclaved plastic flasks into *S. cerevisiae* yeast growth media, which was subsequently identified as BPA [3]. This finding raised the first alarm about BPA’s potential to leach from plastic containers into food and beverages [4,5,6,7], posing a threat to human health. The risks associated with BPA leaching were confirmed in another study, where a sudden increase in meiotic aneuploidy was observed in oocytes from wild-type female mice [8]. The cause of this phenomenon was accidental exposure of the mice to BPA, which had leached from their cages and feeding bottles [8]. The ubiquity of BPA exposure in the world’s population is well documented, with studies detecting BPA in the urine of 95% of tested individuals, with children under six exhibiting higher concentrations [9,10,11,12,13,14].

Concerns about human health arose from the fact that BPA, due to its structural similarity to estrogen, can mimic its hormonal activity, disrupting endocrine signaling even at low doses [15,16,17]. Numerous studies have explored the association between BPA and hormone-related cancers, such as breast, prostate, and ovarian cancers [18]. BPA is suspected to act as a cancer-promoting agent in thyroid and breast cancers [19,20,21,22] and has been linked to the increasing incidence of lung cancer in non-smoking women [23]. BPA has been classified by the International Agency for Research on Cancer as a Group 2A agent, indicating it as a probable human carcinogen. Additionally, BPA exposure has been implicated in brain development defects [24,25], impaired immune function [26] and has been linked to an increased risk of diabetes [27].

BPA is also a prevalent component of microplastics, which are increasingly recognized for their impact on the environment and human health [28,29]. Microplastics have been detected in multiple tissues, including the liver, lungs, spleen, and placenta, as well as in bodily fluids like blood and breast milk [30]. While the full impact of microplastics on human health remains under investigation, multiple studies have suggested significant health risks [29,30]. For instance, microplastics have been implicated in cardiovascular diseases, such as stroke and heart attack [31], and are speculated to contribute to the rising incidence of early-onset colorectal cancer [32]. Despite all the above evidence of the harmful effects of BPA, BPA continues to be used widely, albeit with restrictions, and its use has been banned only in infant products. To address consumer concerns, many manufacturers market products as “BPA-free”. However, these often contain BPA analogs, which may be equally harmful [33,34].

Understanding BPA’s mechanisms of action is a pressing scientific challenge. Beyond its ability to mimic estrogen by binding to estrogen receptors (ESrs), BPA has been associated with oxidative stress [35,36], mitochondrial damage [37,38], and other cellular dysfunctions [39,40]. These effects have been observed in both *Saccharomyces cerevisiae* [41] and mammalian cells, highlighting the evolutionary conservation of BPA’s impact on cellular pathways in eukaryotes. The yeast model system is particularly advantageous for discerning ESr-dependent from ESr-independent BPA effects, as *S. cerevisiae* lacks ESrs [41,42,43,44]. Transcriptional profiling of yeast cells exposed to BPA has revealed extensive gene regulation changes in a dose-dependent manner [44]. However, a significant gap exists in understanding how BPA affects the yeast proteome.

Here, we sought to address this gap by profiling the proteomic response of wild-type and *PDR5*-deleted *S. cerevisiae* strains to BPA exposure. We found that the absence of the ABC multidrug transporter Pdr5, which plays a crucial role in exporting toxic compounds, sensitizes cells to BPA. Our findings show that BPA inhibits yeast growth in a dose-dependent manner, with a more pronounced effect in *PDR5*-deleted cells. Proteomic analysis reveals that BPA induces widespread changes in protein abundance in a dose-dependent manner, highlighting pathways, such as arginine metabolism and mitochondrial protein import. Furthermore, our study underscores both the oxidative stress induced by BPA and the expression of multiple stress-induced proteins upregulated by this compound. These insights advance our understanding of BPA’s cellular impacts, providing a framework for assessing its broader implications for human health.

## 2. Materials and Methods

### 2.1. Materials

The reagents used in this work are commercially available. The *S. cerevisiae* strains used (wild-type and *pdr5*Δ) were purchased from Horizon Scientific (Cambridge, UK). YPD media was from SunriseScientific (Knoxville, TN, USA), and BPA was from MilliporeSigma (Burlington, MA, USA). The protease inhibitors, Y-PER yeast protein extraction reagent, the BCA kit, trypsin protease, and the tandem mass tag (TMTpro) isobaric reagents were from ThermoFisher Scientific (Rockford, IL, USA). Lys-C protease was purchased from Fujifilm Wako (Richmond, VA, USA). Mass spectrometry-grade water and organic solvents were from J.T. Baker (Center Valley, PA, USA). Empore-C18 disks used to make StageTips were from CDSanalytical (Oxford, PA, USA), while Sep-Pak cartridges used in sample preparation (50 mg) were from Waters (Milford, MA, USA).

### 2.2. S. cerevisiae Strains, Growth Conditions, Cell Lysis, and Protein Extraction

The yeast strains used in this work belong to the BY4742 genetic background (*his3*Δ*1*, *leu2*Δ*0*, *lys2*Δ*0*, *ura3*Δ*0*) [45]. *S. cerevisiae* cultures were grown overnight in YPD medium at 24 °C (1% yeast extract, 2% bactopeptone, 2% glucose). The next day, cultures were divided in triplicate and diluted with fresh YPD medium to OD_600_ = 0.15 (ethanol treatment) or OD_600_ = 0.3 (drug treatment). After 6 h, we collected yeast cells by centrifugation at 2000× *g* (2 min), rinsed the cell pellet with 1 mL water, and flash-frozen it in liquid nitrogen. We stored the frozen pellets at −80 °C until sample processing. BPA stock solutions were prepared in ethanol, and the concentration of ethanol in YPD medium was 1% in all the conditions (no treatment, 50 mg/mL, 150 mg/mL, and 300 mg/mL). The growth curve was generated by measuring the optical density of the cell cultures, treated as indicated, at OD = 600 nm every two hours. Cell lysis and protein extraction were performed as previously [46]. Briefly, cell pellets were lysed with the Y-PER yeast protein extraction reagent following the manufacturer’s instructions. Protein concentration was determined using a BCA assay performed according to the manufacturer’s instructions. Proteins were reduced with 5 mM tris(2-carboxyethyl)phosphine (TCEP) for 20 min, alkylated with 10 mM NEM for 20 min (in the dark), and finally quenched with 10 mM dithiothreitol (DTT) for 20 min (in the dark). All reactions were incubated at room temperature [47]. A total of 100 µg of protein from each sample was precipitated by chloroform–methanol precipitation [48].

### 2.3. Protein Digestion, TMT Labeling, and Sample Processing

The samples were digested using Lys-C (overnight at 24 °C) and trypsin (6 h at 37 °C). A total of 1 µg of each enzyme was used per 100 µg of protein. A final volume of 30% acetonitrile was added to each digest, followed by the addition of specified tandem mass tag (TMTpro) labeling reagents [47]. An aliquot of 50 µg of peptide for each sample was labeled with 100 µg of the appropriate TMTpro reagent as follows: wild-type EtOH 126,127n; wild-type 50mg/mL 127c 128n; wild-type 150 mg/mL 128c 129n; wild-type 300 mg/mL 129c 130n; *pdr5*Δ EtOH 130c 131; *pdr5*Δ 50 mg/mL 131c 132n; *pdr5*Δ 150 mg/mL 132c 133n; *pdr5*Δ 300 mg/mL 133c 134n. The samples underwent a one-hour incubation at room temperature. Labeling efficiently was verified before finishing the sample preparation. Briefly, ~1 µg of peptide from each sample was combined and analyzed to verify labeling efficiency (ensuring that it was >97%) [49]. Each sample was treated with hydroxylamine (final concentration of ~0.3%), to quench the labeling reaction, for 15 min. The labeled samples were pooled together 1:1 and desalted with a 50 mg Sep-Pak solid-phase extraction column. Peptide fractionation was executed using basic pH reversed-phase (BPRP) high-performance liquid chromatography (HPLC). A 1260 pump with a 300 Extend C18 column (3.5 μm particles, 2.1 mm ID, and 250 mm in length) from Agilent was used (Lexington, MA, USA). A linear gradient from 5% to 35% acetonitrile in 10 mM ammonium bicarbonate pH 8 with a flow rate of 0.25 mL/min was used to fractionate peptides, and 96 fractions were collected. These 96 fractions were then concatenated and condensed into 24 superfractions [50]. Formic acid 1% was added to the superfractions to acidify them. Each superfraction was vacuum-centrifugated to near dryness and desalted via StageTip. After desalting, each superfraction was dried by vacuum centrifugation and resuspended in 5% acetonitrile and 5% formic acid.

### 2.4. Mass Spectrometry Data Acquisition and Processing

Mass spectrometry data were collected using an Orbitrap Astral mass spectrometer (Thermo Fisher Scientific, San Jose, CA, USA) coupled with an nLC-1200 liquid chromatograph. Peptides were separated on a 100 μm inner diameter microcapillary column packed with ~35 cm of Accucore C18 resin (2.6 μm, 150 Å, Thermo Fisher Scientific). We loaded ~1 μg onto the column. Peptides were separated using a 75 min gradient of 5 to 30% acetonitrile in 0.125% formic acid with a flow rate of 320 nL/min.

The scan sequence began with an Orbitrap MS1 spectrum with the following parameters: resolution 120,000, scan range 350−1350 Th, automatic gain control (AGC) target 200%, maximum injection time 50 ms, RF lens setting 50%, and centroid spectrum data type. FAIMS was enabled with compensation voltages (CVs): −35 V, −45 V, −55 V, −65 V, and −75 V. We selected the top 35 precursors for MS2 analysis, which consisted of HCD high-energy collision dissociation with the following parameters: Astral data acquisition (TMT on), AGC 100%, maximum injection time 30 ms, isolation window 0.4 Th, normalized collision energy (NCE) 35%, and centroid spectrum data type. In addition, unassigned and singly charged species were excluded from MS2 analysis and the dynamic exclusion was set to 15 s.

Spectra were converted to mzXML using MSconvert (v. 3.0.24094) [51], after which database searching included all *S. cerevisiae* entries from UniProt and all protein sequences in that database in reverse order. Searches were performed using a 50 ppm precursor ion tolerance and a product ion tolerance of 0.02 Da to maximize sensitivity, in conjunction with Comet database searching and linear discriminant analysis (LDA) [52,53]. TMTpro tags on lysine residues and peptide N-termini (+304.207 Da) and carbamidomethylation of cysteines (+57.021 Da) were set as static, whereas oxidation of methionine residues (+15.995 Da) was set as a variable modification. Peptide-spectrum matches (PSMs) were adjusted to a 1% false discovery rate (FDR), and filtering thereof was performed using LDA to assemble the dataset further to achieve a final protein-level FDR of 1% [54]. Once completed, the proteins were quantified by summing reporter ion counts across matching PSMs [55]. PSMs with a signal-to-noise value < 1000 or resolving power < 40,000 for reporter ions were omitted from further analysis. Reporter ion intensities were corrected for the isotopic impurities of the TMT reagents according to the manufacturer’s specifications. The signal-to-noise (S/N) measurements of peptides assigned to each protein were summed and normalized such that the sum of the signal for all proteins in each channel was the same, thereby accounting for unequal protein loading (i.e., column normalization was performed). Finally, each protein abundance measurement was represented as a percentage of the total, in that the summed S/N for that protein across all channels was 100, thus providing a relative abundance (RA) measurement. Proteins were considered significantly changing if they met the fold change threshold of|log_2_ ratio| > 0.5 and a *p*-value of less than 0.05.

## 3. Results

### 3.1. BPA Inhibits S. cerevisiae Cell Growth

**Figure 1:**
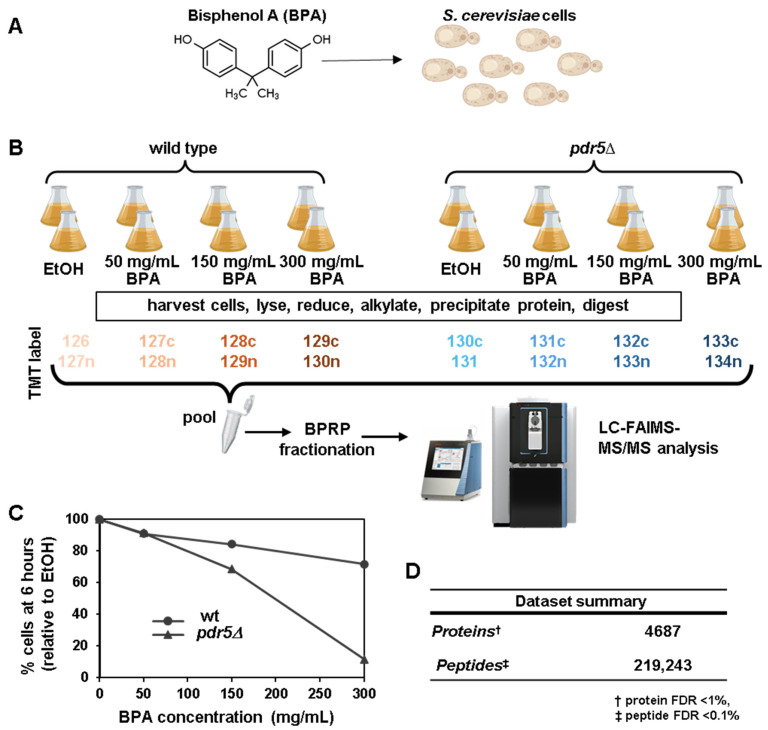
Experimental workflow, dataset summary, and the effect of bisphenol A (BPA) on cellular growth after six-hour treatment. (**A**) Wild-type and *pdr5*∆ *S. cerevisiae* cells were grown in duplicate to exponential phase (24 °C) and treated with the indicated BPA concentrations or ethanol (EtOH) as a control for six hours. (**B**) Cells were harvested and processed for mass spectrometry analysis. In brief, yeast cells were lysed, and total protein was extracted and digested. The subsequent peptides were labeled with tandem mass tag (TMTpro) reagents, as indicated, pooled 1:1, and fractionated by basic pH reversed-phase (BPRP) HPLC prior to mass spectrometry analysis. This panel was assembled, in part, using Biorender.com. (**C**) Percentage of cells at 6 h treated with the indicated BPA concentration compared to EtOH-treated cells. (**D**) Dataset summary.

To elucidate the cellular pathways affected by bisphenol A (BPA) (Figure 1A), we profiled the proteome of wild-type (wt) and *pdr5*Δ *S. cerevisiae* cells following six hours of treatment with low (50 mg/mL), medium (150 mg/mL), and high (300 mg/mL) concentrations of BPA using ethanol (EtOH) as a control. We employed isobaric-tag-based quantitative proteomics (Figure 1B), leveraging a single TMTpro18-plex experiment with duplicates for each condition. The experimental setup involved exposing both yeast strains to four different conditions: a control (EtOH) and three increasing concentrations of BPA (50, 150, and 300 mg/mL). Following exposure, the cells undergo a series of processing steps for mass spectrometry analysis, including harvesting, lysis, reduction, alkylation, protein precipitation, and digestion [45]. The resulting peptides are then labeled using Tandem Mass Tag (TMT) isobaric labels, with each condition assigned a specific label, enabling multiplexed analysis [56]. TMT labeling is a crucial aspect of this methodology, allowing for simultaneous analysis of multiple samples. In this experiment, different isotopic variants of the TMT reagents were used to label peptides from each experimental condition. These isotopic variants have identical chemical properties, but differ slightly in mass due to the incorporation of different combinations of 13C and 15N isotopes [57]. When peptides from different samples are labeled with these isotopically distinct tags and then mixed, they behave identically during chromatographic separation and ionization. But, upon fragmentation in the mass spectrometer, the tags release reporter ions of different masses, each corresponding to a specific experimental condition. The relative intensities of these reporter ions reflect the relative abundances of the peptides (and thus proteins) across the different samples. This allows for precise relative quantification of proteins between the wild-type and *pdr5*Δ yeast strains under various BPA concentrations. The labeled samples were subsequently pooled and fractionated using basic pH reversed-phase (BPRP) chromatography to reduce sample complexity [58]. Finally, the fractionated samples were analyzed using liquid chromatography–field asymmetric waveform ion mobility spectrometry–tandem mass spectrometry (LC-FAIMS-MS/MS). This experimental design allows for a comparative analysis of the wild-type and *PDR5* deleted yeast proteomes under varying BPA concentrations. The use of isobaric tagging and multiplexing techniques enhances experimental efficiency and reduces variability, while the fractionation step increases proteome coverage, altogether providing a powerful approach to understanding the molecular mechanisms underlying BPA toxicity and defense in yeast. This analysis quantified over 75% of the annotated yeast proteome, specifically 4687 proteins (at a 1% false discovery rate), from 219,243 unique peptides (Figure 1D, Table S1).

The BPA concentrations of 50 mg/mL and 300 mg/mL were selected based on a prior transcriptomic study in the wild-type strain belonging to the genetic background BY4742 [44], the same which we used. Additionally, we included an intermediate concentration of 150 mg/mL as we also analyzed the proteome of a *pdr5*Δ strain, which lacked the ABC multidrug transporter Pdr5, a key player against xenobiotic compounds [59]. In parallel to the proteome analysis, we collected samples to measure the growth rates of wild-type and *pdr5*Δ cells under these conditions (Figure S1A,B). At 50 and 300 mg/mL BPA, wild-type cell growth was inhibited by approximately 10% and 30%, respectively, after 6 h (Figure 1C). In contrast, *pdr5*Δ cells showed growth inhibition of 10% at 50 mg/mL and a dramatic 90% at 300 mg/mL (Figure 1C). The intermediate concentration (150 mg/mL) inhibited wild-type growth by 15% and *pdr5*Δ growth by 30% (Figure 1C and Figure S1A,B). These findings confirm that BPA inhibited *S. cerevisiae* growth in a dose-dependent manner, consistent with previous studies [41,44]. Furthermore, we observed that *pdr5*Δ cells showed greater sensitivity to BPA, suggesting that Pdr5 plays a protective role in exporting BPA from yeast cells.

### 3.2. BPA Induces Proteome Changes in a Dose-Dependent Manner

**Figure 2:**
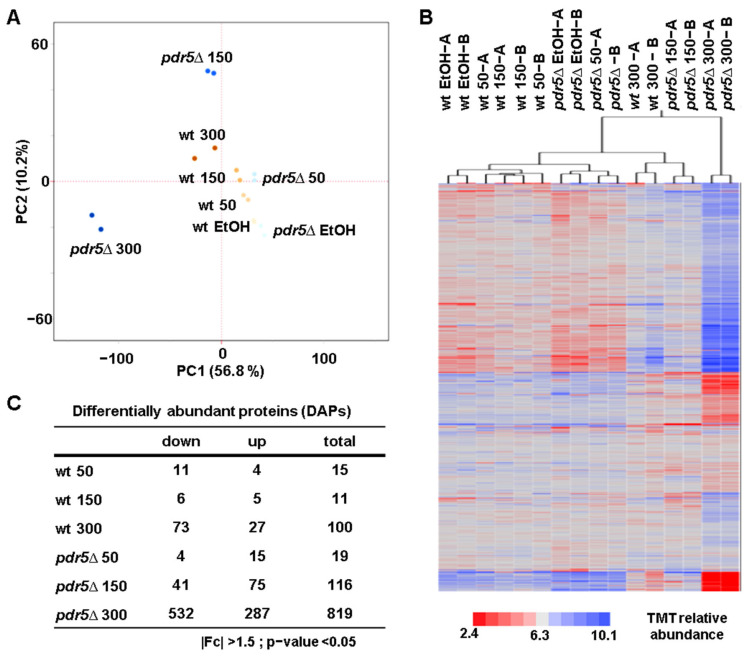
Principal component analysis (PCA), hierarchical clustering analysis (HCA), and differentially abundant proteins (DAPs) in wild-type and *pdr5*Δ cells treated with BPA. (**A**) PCA of the dataset illustrates the clustering of the replicates. (**B**) HCA of the TMT relative abundance (TMT RA) for the 4687 proteins quantified across the 16 TMT channels. Duplicates of each condition are indicated as A and B. (**C**) The table summarizes the differentially abundant proteins (DAPs) in the two yeast strains, wt and *pdr5*Δ, treated with the indicated BPA concentrations compared to the control (EtOH-treated) strains.

To assess the overall proteome changes, we performed principal component analysis (PCA) and hierarchical clustering analysis (HCA). PCA revealed tight clustering of replicates, with PC1 explaining over 56% of the variance and PC2 accounting for 10% (Figure 2A). Notably, *pdr5*Δ cells treated with 150 mg/mL and 300 mg/mL of BPA displayed distinct clustering, likely due to higher intracellular BPA accumulation. HCA, conducted using Euclidean distance and Ward linkage, showed similar clustering patterns (Figure 2B). Interestingly, wild-type cells treated with 50 mg/mL of BPA clustered with those treated with 150 mg/mL, suggesting similar proteomic responses at these lower BPA concentrations. However, wild-type cells treated with 300 mg/mL of BPA clustered near *pdr5*Δ cells treated with 150 mg/mL reflecting more pronounced proteome alterations. Lastly, *pdr5*Δ cells treated with 300 mg/mL of BPA formed a distinct cluster, exhibiting even more significant alterations in protein abundance.

Next, we identified differentially abundant proteins (DAPs) as those with a |log2 fold change| > 0.5 and an unadjusted *p*-value < 0.05 (Figure 2C). The number of DAPs increased with BPA concentration in both wild-type and *pdr5*Δ cells. Consistent with HCA, the magnitudes of proteomic changes induced by 50 mg/mL and 150 mg/mL BPA in wild-type cells were similar. As expected, *pdr5*Δ cells exhibited a greater number of DAPs across all concentrations, supporting the hypothesis of elevated intracellular BPA levels compared to wild-type cells due to the absence of Pdr5.

### 3.3. BPA Exposure Affects the Levels of the Pleiotropic Drug Resistance (PDR) Proteins

We confirmed the absence of Pdr5 in *pdr5*Δ cells by measuring its protein abundance in our proteomic experiment. We observed some residual signal in *pdr5*Δ cells likely due to interference (Figure S2A) [60]. Interestingly, wild-type cells exposed to BPA exhibited a dose-dependent increase in Pdr5, supporting its role in mediating BPA resistance. These data align with the higher growth inhibition and the stronger proteome changes observed in *pdr5*Δ cells (Figure 1C and Figure 2C).

The PDR family of proteins is responsible for multidrug resistance and includes several members [61], in addition to Pdr5, which may contribute to BPA resistance. We quantified seven PDR members in our proteomic experiment (Figure S2B): the transcription factors Pdr1, Pdr3, and Pdr8; the transporters Pdr12 and Pdr15; and the phosphatidylinositol transfer proteins Pdr16 and Pdr17. Notably, Pdr16 abundance increased in both wild-type and *pdr5*Δ cells in a dose-dependent manner. In contrast, Pdr15 abundance increased only in *pdr5*Δ cells, suggesting that it may compensate for the absence of its paralog Pdr5. However, Pdr12 abundance decreased in *pdr5*Δ cells with an increasing BPA concentration. These results highlight the dose-dependent regulation of specific PDR proteins by BPA and the intricate regulatory network among them in response to this compound.

### 3.4. BPA Induces Arginine Biosynthesis and Glucose Transporters While Downregulating Mitochondrial Proteins in Wild-Type Cells

**Figure 3:**
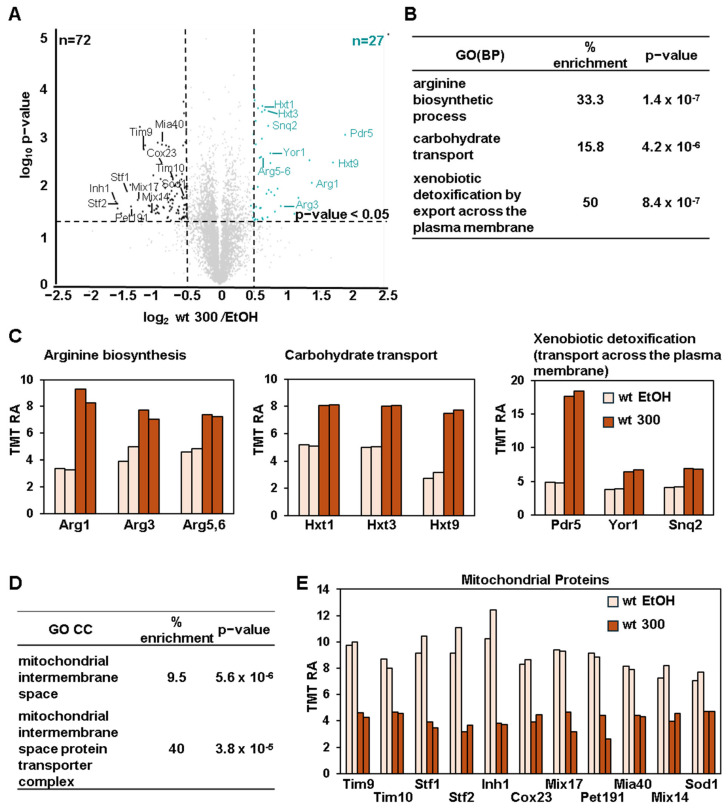
Proteome-wide profiling of differentially abundant proteins in wild-type cells after treatment with 300 mg/mL BPA. (**A**) The volcano plot illustrates differentially abundant proteins (i.e., |log_2_ ratio| > 0.5, and *p*-value < 0.05) in wild-type cells after treatment with 300 mg/mL BPA. Proteins highlighted in (**C**,**E**) are labeled. (**B**) The top gene ontology (GO) biological processes (BP) terms associated with the proteins that are increasing in (**A**). (**C**) Bar graphs illustrate the TMT relative abundance (RA) of the classes of proteins in (**B**). (**D**) The top GO cellular component (CC) terms associated with proteins with decreased abundance after BPA treatment. (**E**) TMT relative abundance measurements of mitochondrial proteins with decreased abundance in (**A**).

As only a small number of DAPs were identified at lower doses of BPA (50 and 150 mg/mL) in wild-type cells (Figure 2C). We focused our analysis on wild-type cells treated with 300 mg/mL BPA. We identified 27 significantly upregulated and 72 downregulated proteins compared to EtOH-treated cells (Figure 3A). Gene ontology (GO) analysis of the upregulated proteins revealed enrichment of pathways related to arginine biosynthesis, carbohydrate transport, and xenobiotic detoxification (Figure 3B). Specifically, the arginine biosynthesis enzymes Arg1, Arg3, and Arg5,6 showed increased abundance (Figure 3C, left). Additionally, low-affinity glucose transporters Hxt1, Hxt3, and Hxt9 were upregulated (Figure 3C, middle). Low-affinity glucose transporters are usually upregulated when glucose levels in the medium are high, suggesting that BPA altered glucose uptake or metabolism. BPA also increased the abundance of ABC transporters Snq2 and Yor1, alongside Pdr5, indicating their potential roles in BPA resistance (Figure 3C, right). As the upregulation of Pdr5 induced by BPA is much higher compared to that of Snq2 and Yor1, it is likely that the deletion of *PDR5* would have a strong effect on intracellular BPA accumulation. These data indicated that deleting these two genes, along with *PDR5*, could further enhance the effects of BPA on cellular functions and on the proteome compared to cells in which only *PDR5* has been deleted.

Conversely, BPA downregulated a set of mitochondrial proteins, specifically those in the mitochondrial intermembrane space and those constituting the mitochondrial intermembrane space protein transporter complex (Figure 3D). These proteins included the transporters Tim9, Tim10, and the assembly factors Mia40 and Cox23 (Figure 3E). Reduced levels of these proteins suggest that BPA could disrupt mitochondrial protein import, a process critical for mitochondrial function.

### 3.5. Proteomic Changes in pdr5Δ Cells Indicate Oxidative Stress

**Figure 4:**
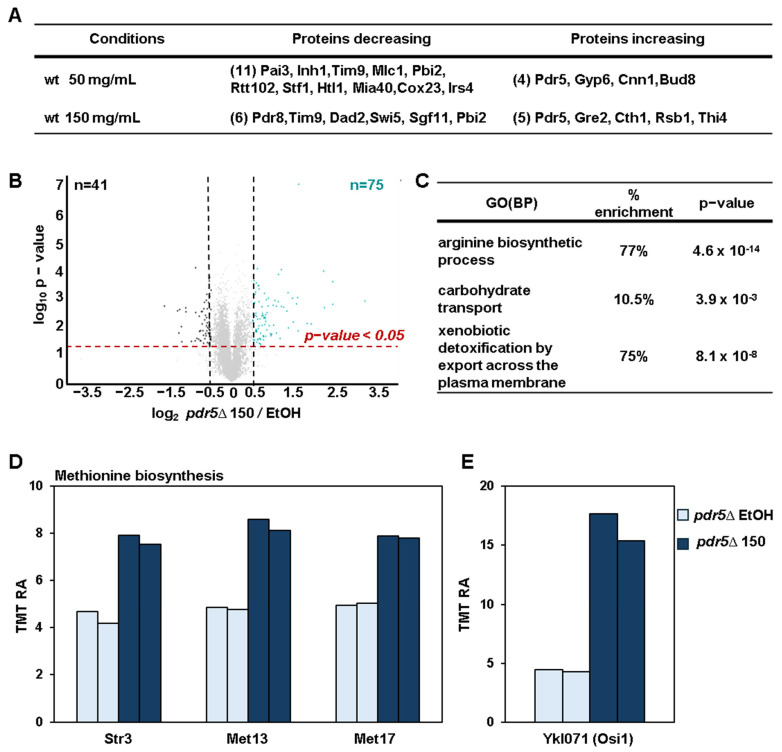
Proteins changing with lower doses of BPA in wild-type cells and proteome-wide profiling of differentially abundant proteins in *pdr5*∆ cells treated with 150 mg/mL BPA. (**A**) The table summarizes the differentially abundant proteins in wild-type cells treated with 50 and 150 mg/mL of BPA compared to the EtOH condition. (**B**) The volcano plot illustrates differentially abundant proteins (i.e., |log_2_ ratio| > 0.5, and *p*-value < 0.05) in *pdr5*Δ cells after treatment with 150 mg/mL BPA compared to *pdr5*Δ cells treated with EtOH. (**C**) The top gene ontology (GO) biological processes (BP) terms associated with the proteins increasing after BPA treatment in *pdr5*Δ cells. Bar graphs illustrate the TMT relative abundance (RA) of proteins increasing in *pdr5*Δ cells involved in (**D**) methionine metabolism and of (**E**) the protein Ykl071 (Osi1, oxidative stress-induced protein 1).

As in wild-type cells, only a small number of DAPs were identified at the lowest dose of BPA (50 mg/mL) in wild-type cells (Figure 4A). We identified 75 and 41 proteins with higher and lower abundance, respectively (Figure 4B), from our analysis of *pdr5*Δ cells treated with 150 mg/mL BPA compared to the EtOH-treated control cells. Here, we have omitted the comparison with the 300 mg/mL dose as it caused near-complete growth inhibition of *pdr5*Δ cells (Figure 1C). GO analysis highlighted similar enrichment patterns observed for wild-type cells treated with 300 mg/mL BPA, including arginine biosynthesis, carbohydrate transport, and detoxification pathways (Figure 4C). Additionally, we observed upregulation of methionine biosynthesis enzymes (e.g., Str3, Met13, and Met17) and of Osi1 (oxidative stress-induced protein 1), which are both markers of oxidative stress (Figure 4D,E). These findings suggest that BPA induces oxidative stress, which is consistent with previous studies in yeast and mammalian cells [35,41].

### 3.6. BPA-Induced Stress and Cellular Damage to S. cerevisiae Cells

In agreement with the observed dose-dependent cell growth inhibition by BPA, several BPA-responsive proteins provided insights into the stresses and cellular damage induced by this compound. For instance, Rsb1, a transporter activated by membrane stress and by glycerophospholipid asymmetry [62], was upregulated in a dose-dependent manner (Figure S3). Similarly, stress-response proteins Gre2 [63] and Tsv1 [64] increased with BPA exposure, indicating the presence of a cellular response to stress (Figure S3). Dld3 [65], associated with mitochondrial dysfunction, was also of relatively higher abundance (Figure S3), corroborating our earlier observations of disrupted mitochondrial protein import. Yhi9 [66] and Iml2 [67], for which upregulation was particularly evident in *pdr5*Δ cells treated with 150 mg/mL, were involved, respectively, in the endoplasmic reticulum unfolded protein response and in the clearance of inclusion bodies that form during protein misfolding stress (Figure S3). These findings demonstrated that BPA exposure triggered a wide range of cellular stress responses and proteome changes, which can serve as a valuable resource and starting point for uncovering BPA’s mechanisms of action.

## 4. Discussion

Here, we investigated the impact of different doses of BPA on cell growth and on the proteome of *S. cerevisiae* wild-type and *pdr5*Δ strains. Yeast cells, which naturally lack estrogen receptors (ESr), offer a distinct advantage for studying BPA. By analyzing their response to BPA, we can identify BPA targets that operate independently of the ESr pathway. As the effects of BPA on eukaryotic cells appear to be conserved [42], our data could help distinguish between ESr-mediated and non-ESr-mediated BPA-dependent effects in higher eukaryotes.

Consistent with previous studies in *S. cerevisiae* [41,44], we observed that BPA inhibited cell growth in a dose-dependent manner. Additionally, we demonstrated that the deletion of *PDR5* sensitizes yeast cells to BPA, suggesting that this ABC transporter is required to export BPA out from yeast cells. In agreement with this, BPA increased the protein abundance of Pdr5 in a dose-dependent manner in the wild-type strain. Furthermore, the proteome-level changes were more pronounced in *pdr5*Δ cells compared to wild-type cells, likely reflecting the higher BPA uptake in *pdr5*Δ cells.

In addition to Pdr5, we observed that other ABC transporters, such as Snq2 and Yor1, were also upregulated in a BPA-dependent manner. These data suggest that these transporters may contribute to BPA resistance in yeast cells. To comprehensively assess the impact of BPA on yeast cells, it may be advantageous to investigate further cells with combined deletions of *PDR5* and *SNQ2* or *YOR1*. Such multiple deletions are likely to result in higher intracellular concentrations of BPA, thereby amplifying its effects compared to wild-type cells. We speculate that at lower doses of BPA, these transporters’ actions do not significantly alter the proteome or the growth of wild-type cells. Our data suggest that the role of ABC transporters in exporting BPA outside the cells is conserved, as this class of transporter has been also implicated in removing BPA from human cells [68].

One unexpected finding from our proteome analysis was that BPA induced an increase in the protein abundance of glucose transporters in both wild-type and *pdr5*Δ cells. Interestingly, the glucose transporters that were upregulated by BPA were specifically those with low affinity, such as Hxt1, Hxt3, and Hxt9. These transporters are usually upregulated when yeast cells sense more glucose in the medium [69]. An analysis of the literature revealed that BPA increases the abundance of the glucose transporter GLUT1 (Hxt1 homolog) in rat’s placenta and in human trophoblasts [70], as well as GLUT4 in mouse adipocytes [71]. These data suggest that BPA may influence glucose uptake with a mechanism that is likely evolutionarily conserved. As an increase in glucose transporters is observed in *S. cerevisiae*, which lacks estrogen receptors (ESRs), this function of BPA is likely ESr-independent. The increase in glucose transporters could contribute to the observed link between BPA and diabetes development [27]. However, the mechanism underlying the upregulation of glucose transporters remains unclear.

We also observed an increase in enzymes involved in arginine biosynthesis in both wild-type and *pdr5*Δ cells and an increase in enzymes involved in methionine biosynthesis in *pdr5*Δ cells. Both arginine and methionine biosynthesis have been shown to be upregulated in *S. cerevisiae* in response to oxidative stress [72,73], suggesting that *S. cerevisiae* cells treated with BPA may be experiencing this type of stress. Consistent with this finding, Osi1 or oxidative stress-induced protein 1, a known marker of oxidative stress [74], was one of the proteins with the highest fold change in *pdr5*Δ cells treated with BPA. We hypothesized that the increase in methionine biosynthesis enzymes was not evident in wild-type cells, as significantly less BPA accumulated in these cells compared to *pdr5*Δ cells. Our proteome analysis suggests that BPA induces oxidative stress in *S. cerevisiae*. To validate this observation, it is essential to use standard methods to measure oxidative stress, such as assessing ROS levels or detecting oxidatively modified proteins. It is worth noting that these methods have been employed in previously published studies. Indeed, several studies have reported that BPA can induce oxidative stress in both *S. cerevisiae* and mammalian cells [35,36,41]; however again the mechanisms underlying this effect remain uncharacterized.

Proteomic analysis has been performed in multiple model systems, such as human, mice, rat, and zebrafish cells. Exposure to BPA has led to the alteration of proteins involved in multiple signaling pathways, such as pathways associated with tumor progression [75], development and metabolism [76], and endocrine and reproductive signaling [77,78]. Furthermore, multiple studies have identified alterations that indicate the presence of toxicity and stresses, in particular oxidative stress [35,79,80].

One of the most interesting findings from our analysis was the downregulation of a specific set of mitochondrial proteins, particularly those located in the intermembrane space. Mitochondrial dysfunction has been shown to be induced by BPA in multiple model systems [37,38,41], but the underlying mechanisms thereof remain undiscovered. Our data suggest that mitochondrial dysfunction in *S. cerevisiae* cells may be a result of defects in the process of mitochondria protein import.

A previous transcriptomic analysis examining the effects of BPA on the yeast transcriptome, conducted using the same yeast background as in our study [44], revealed findings that align with our proteomic analysis. Specifically, it reported the upregulation of Pdr5 and Pdr16 and the downregulation of mitochondrial proteins. Additionally, we observed a downregulation of glucose transporters induced by low glucose levels, which is consistent with our observation of low affinity glucose transporter upregulation.

Overall, our proteome analysis, the first on the effects of BPA on the proteome of *S. cerevisiae*, suggests the cellular pathways that can be affected by this compound. Additionally, we identified hundreds of differentially abundant proteins regulated at the protein level by BPA. Among these, we highlighted a few, such as Yhi9, which is involved in the unfolded protein response, and Dld3, which is upregulated in response to mitochondrial dysfunction. We hypothesize that many of these proteins regulated in a BPA-dependent manner will help to eventually fully elucidate the mechanism of action of this compound and will guide future studies to better comprehend its impact on human health.

## Data Availability

RAW files will be made available upon request, in addition to the data that have been deposited to the ProteomeXchange Consortium via the PRIDE [81] partner repository with the dataset identifier. Project accession: PXD058913.

## Supplementary Materials

The following supporting information can be downloaded at: https://www.mdpi.com/article/10.3390/microorganisms13010114/s1, Figure S1: Yeast growth curves; Figure S2: Protein abundance profile of Pdr5 and of other members of the PDR protein network; Figure S3: Example proteins that change in a BPA dose-dependent manner; Table S1: Proteins quantified in the experiment.
