# Supplementary material for: Comprehensive Analysis of the Proteome of S. cerevisiae Wild-Type and pdr5Δ Cells in Response to Bisphenol A (BPA) Exposure
Source: Microorganisms. 2025 Jan 8;13(1):114. doi: 10.3390/microorganisms13010114 (PMC11767658; doi:10.3390/microorganisms13010114)
Supplement: Supplementary file 1 [file microorganisms-13-00114-s001.zip › microorganisms-3405645 Supplementary Figures_JP.pdf]

microorganisms

MDPI

Supplementary materials for “Comprehensive Analysis of the Proteome of *S. cerevisiae* Wild-Type and *pdr*5Δ Cells in Response to Bisphenol A (BPA) Exposure”

Figure S1:

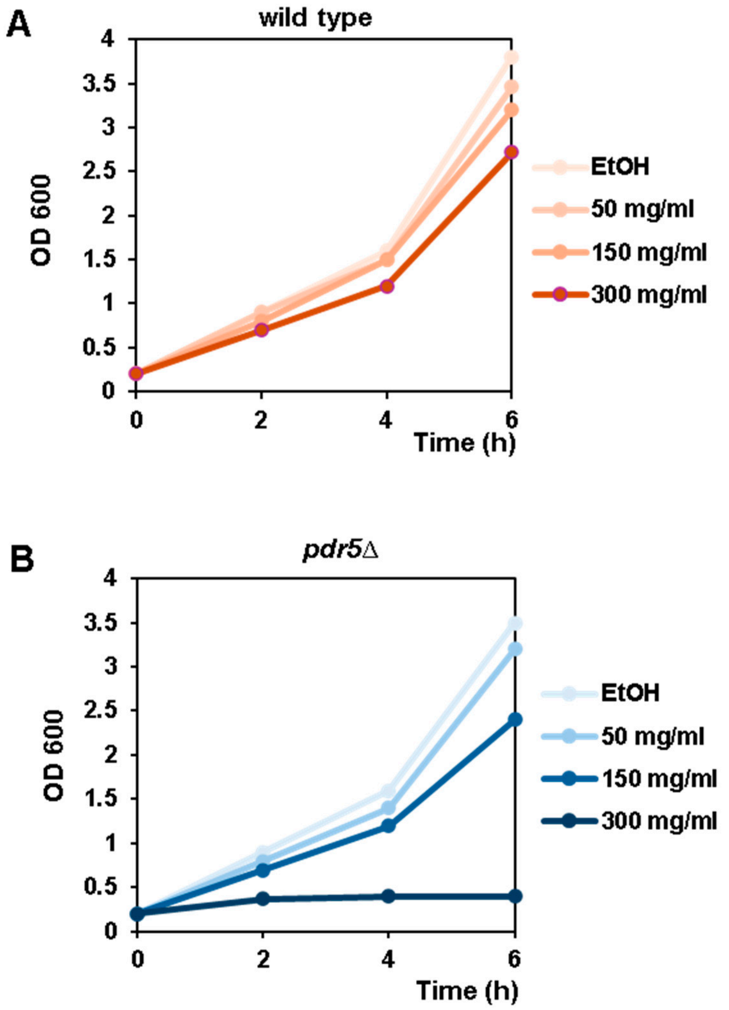

Figure S1. Yeast growth curves. Growth curves of (**A**) wild-type and (**B**) *pdr5D* cells in the presence of EtOH (control) or BPA at the indicated concentrations.

Figure S2:

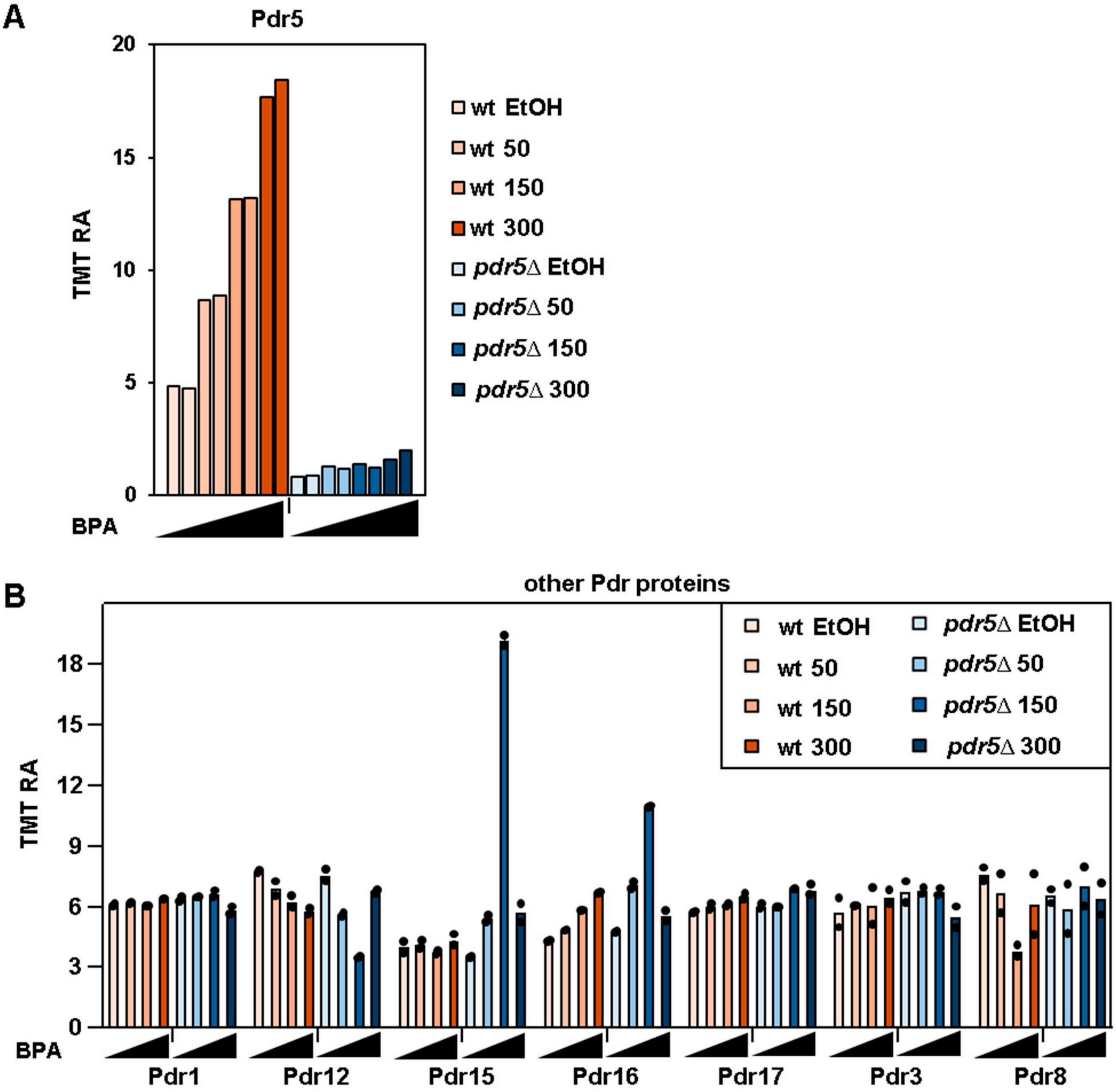

Figure S2. Protein abundance profile of Pdr5 and of other members of the PDR protein network. Bar graphs illustrating the TMT relative abundance (RA) of Pdr5 (**A**) and of all the detected Pdr proteins (**B**) in the proteomic experiment.

Figure S3:

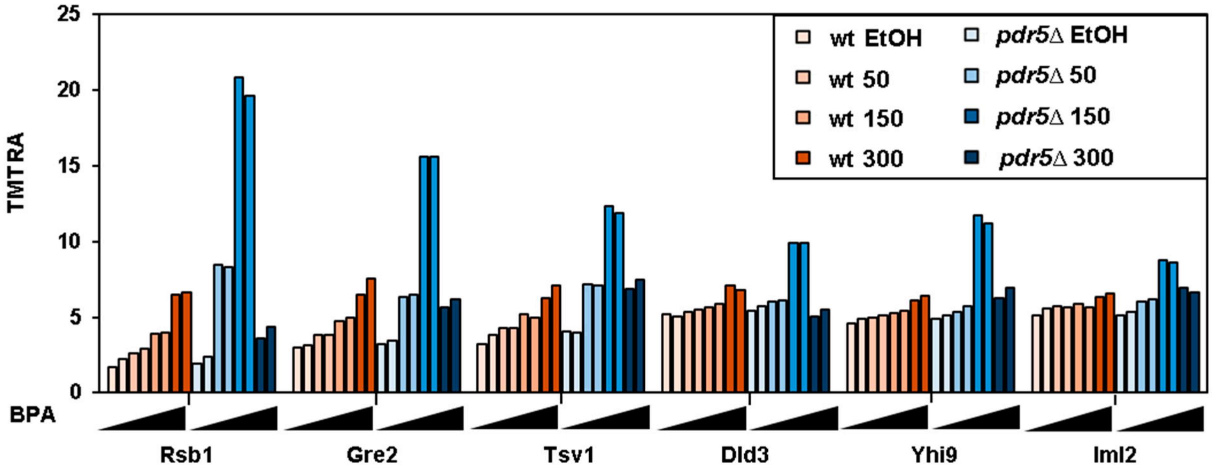

Figure S3. Example proteins that change in a BPA dose-dependent manner. Bar graphs illustrate the TMT relative abundance (RA) of proteins increasing in a BPA-dependent manner such as, Rsb1 (sphingoid long-chain base transporter), Gre2 (3-methylbutanal reductase), Tsv1/Ycr061w (trans-membrane protein vital for stress response), Dld3 (2-hydroxyglutarate transhydrogenase), Yhi9 (member of the PhzF superfamily), and Iml2 (protein required for the clearance of inclusion bodies).
